# META-SYNTHESIS: OLDER ADULT SURVIVORS’ RESILIENCE

**DOI:** 10.1093/geroni/igz038.932

**Published:** 2019-11-08

**Authors:** Roberta R Greene, Harriet Cohen, Nancy A Greene, Shira Hantman

**Affiliations:** 1 University of Texas at Austin, Austin, Texas, United States; 2 Jewish Family Service of Dallas, Dallas, Texas, United States; 3 Johnson C. Smith University, Charlotte, North Carolina, United States; 4 Tel Hai College, Upper galilee, Israel, Israel

## Abstract

This paper presents the results of a meta synthesis of 8 qualitative studies that examined resilience among 270 older adult survivors following potentially traumatic adverse events (Bonanno, 2004). The primary data involved information about survivors’ critical events during the Holocaust , the Cambodian genocide, and the discriminatory practices of the Jim Crow U.S. South. A narrative approach to gerontology was used to collect and analyze the original data. known as a constant comparison data analysis ( Glaser & Strauss, 2009; Miles & Huberman, 1994). This allowed for the understanding of risks such as such as discrimination, imprisonment, genocide, and possible loss of life and the development of themes of resilience at the personal, interpersonal, sociocultural, and societal levels (Kenyon & Randall, 2001) The meta synthesis of secondary data involved coding the original findings, grouping them into descriptive themes, and generating new overarching analytical themes (Thomas & Harden, 2008). The most prevailing theme at the personal-level (internal feelings) was overcoming the grief of losing a loved one. Interpersonal-level themes (relationships between people) were related to staying connected to family. Sociocultural-level themes (the beliefs and mores of the time) centered on making meaning of a critical event within its historical context Societal themes (government and institutions) themes were indicative of being able to contribute or influence one’s current community. Implications for clinical practice and policy formation are provided.

